# Exploring the scope of inspiratory muscle training in difficult weaning: reflections on the multicentre RCT

**DOI:** 10.1186/s40560-024-00768-6

**Published:** 2025-03-10

**Authors:** Sireesha Chilakapati, Jyothi Koteswara Rao, Bharat Paliwal

**Affiliations:** 1 Department of Anaesthesiology and Critical Care, AIIMS, Phase II, Basani Industrial Area,, Jodhpur, 342005 Rajasthan India; 2 Department of Anaesthesiology and Critical Care, AIIMS, Jodhpur, India

**Keywords:** Inspiratory muscle training, Maximal inspiratory pressure, Difficult-to-wean, Mechanical ventilation, Respiratory muscle strength, Extubation prediction

## Abstract

We commend the authors for their insightful study on inspiratory muscle training (IMT) in mechanically ventilated patients with difficult weaning, highlighting the robust use of maximum inspiratory pressure (MIP) as a key outcome. We suggest that a lower baseline maximum inspiratory pressure cutoff could better target patients with significant inspiratory dysfunction, improving the study's precision. Additionally, alternative imputation techniques, such as multiple imputation, could strengthen the handling of missing data. While the sample size calculation was appropriate, the unbalanced group sizes raise concerns about generalisability. Future research could benefit from subgroup analyses, individual response curves, and further investigation into the unexpected adverse effects observed in the low-intensity group to refine the inspiratory muscle training protocols.

## To The Editor

We commend the authors on their insightful study, “Impacts of Three Inspiratory Muscle Training Programs on Inspiratory Muscle Strength and Endurance Among Intubated and Mechanically Ventilated Patients with Difficult Weaning: A Multicentre Randomised Controlled Trial” [1]. The study addresses an important clinical challenge in mechanically ventilated patients, and it provides valuable data on the effectiveness of different inspiratory muscle training (IMT) modalities. The use of maximum inspiratory pressure (MIP) as a clinically relevant outcome is particularly commendable, as it adds robustness to the evaluation of respiratory muscle performance. However, while the study contributes significantly to the understanding of IMT protocols, we would like to suggest a few points for further consideration.

Maximal inspiratory pressure (MIP) is a key measure of respiratory muscle strength, particularly the diaphragm, and plays a crucial role in assessing a patient's ability to wean off mechanical ventilation. MIP measures the maximum pressure a patient can generate during a forceful inspiration and provides insight into the strength of the diaphragm and other inspiratory muscles. It is widely used to predict weaning outcomes, with studies suggesting that MIP values more negative than −30 cm H2O indicate a likely successful weaning, while values higher than −20 cm H2O may predict failure [2]. However, MIP has limitations, especially in uncooperative patients or those with poor voluntary effort, as it requires active participation to generate accurate readings. Moreover, MIP reflects diaphragmatic strength primarily during deep inspiration rather than normal breathing, limiting its specificity as a weaning predictor.

To enhance the predictive value of MIP, researchers have explored alternative indices. One such method is the ratio of occlusion pressure (P0.1) to MIP [3], which combines respiratory drive and muscle strength to provide a more reliable predictor of extubation success. Another approach is the use of sustained maximal inspiratory pressure (SMIP) [4], which assesses the ability to maintain maximal inspiratory pressure over time, and has shown higher sensitivity and specificity than MIP alone. Given the diaphragm’s critical role in unassisted breathing, these alternative measures provide a more comprehensive assessment of diaphragmatic function, leading to better predictions of weaning outcomes and extubation success in ICU patients. This refined approach aims to overcome the limitations of traditional MIP testing and offers a more accurate evaluation of a patient’s readiness for independent breathing.

Since the study's sample had a mean baseline MIP of 49.7 ± 17.4 cmH2O, which does not indicate severe inspiratory muscle weakness, using a lower MIP cutoff could have better targeted patients with significant inspiratory dysfunction. This would reduce sample heterogeneity and more accurately assess the effects of IMT on those who need it most. Implementing a cutoff of MIP < −30 cmH2O or MIP < −36 cmH2O, as recommended by De Jong et al. [5] and Tzanis et al.[6], respectively, could help identify difficult-to-wean patients more likely to benefit from IMT, thereby improving the study's precision and outcomes.

The use of linear regression models in this study is noteworthy, as it effectively adjusts for key variables such as study center, baseline MIP, respiratory pathology, and length of mechanical ventilation (MV), helping to control for potential confounders. Additionally, the intent-to-treat (ITT) approach preserves the integrity of randomisation, which is a strong methodological choice. However, while the last observation carried forward (LOCF) strategy for handling missing data is commonly used, it has limitations, as it may introduce bias, particularly if there are underlying trends over time that are not captured. Exploring alternative imputation techniques, such as multiple imputation, could provide more robust and reliable results [7].

The study’s sample size calculation of 88 participants aimed for 80% power to detect a 2 cmH2O difference in maximal inspiratory pressure (MIP) among groups, with projected increases in MIP of 12 cmH2O for the MI group and around 9.7 cmH2O for the HI and LI groups. This adequately supports detecting meaningful changes while applying a Bonferroni correction for a type I error rate of 0.025. However, the final analysis included 89 participants distributed in an unbalanced 2:1:1 ratio. This allocation, with a larger proportion of participants in the MI group compared to the HI and LI groups, may have introduced challenges in drawing equitable comparisons between the groups. The smaller sample sizes in the HI and LI groups not only limit the ability to detect statistically significant differences, but may also increase the variability of results within these subgroups. This imbalance could affect the precision of effect size estimates for these groups, thereby reducing the generalisability of the findings. Moreover, any unexpected outcomes, such as adverse events, in the smaller groups could disproportionately influence the results, highlighting the importance of cautious interpretation in subgroup analyses. The MI protocol’s daily MIP calibration suggests a better adaptation to individual variability. Further analysis could stratify by baseline MIP to determine if the MI protocol benefits diverse patient subgroups. Notably, serious adverse effects like bradycardia in the LI group, though infrequent, indicate a need to explore potential cardiovascular implications through methods like echocardiograms during inspiratory muscle training (IMT). Future trials with larger cohorts may enhance insights into these rare events.

Addressing these aspects in future research could not only strengthen the current findings, but also lead to more personalised and effective treatment strategies for difficult-to-wean patients undergoing inspiratory muscle training. We recommend that the authors consider these points to refine their methodology and enhance the clinical relevance of their work.

## Data Availability

No datasets were generated or analysed during the current study.

## Abbreviations

RCT: Randomised controlled trial
IMT: Inspiratory muscle training
MIP: Maximum inspiratory pressure
SMIP: Sustained maximal inspiratory pressure
ICU: Intensive care unit
MV: Mechanical ventilation
ITT: Intent to treat
LOCF: Last observation carried forward
MI: Mixed intensity
HI: High intensity
LI: Low intensity
ECG: Electrocardiogram
